# Alcohol-related expectations and risky drinking in young adult Czechs

**DOI:** 10.1186/1940-0640-7-S1-A37

**Published:** 2012-10-09

**Authors:** Hana Sovinova, Ladislav Csemy, Bohumir Prochazka

**Affiliations:** 1 National Institute of Public Health, Prague, Czech Republic

## 

The aim of the study was to analyze the structure of expectations linked to alcohol consumption of low-risk drinkers compared with high-risk drinkers. Data from a questionnaire-based survey performed on a representative sample of 2221 persons (51.4% were men) aged 18 to 39 (average age, 29.9) were used for the analysis. The validated Czech version of the Alcohol Use Disorders Identification Test was employed to define low- and high-risk drinkers. Respondents scoring >16 were deemed to be high-risk consumers. To measure alcohol expectancies, we used a 14-item scale: seven items represented pleasurable and socially positive expectancies, and the seven remaining items represented negative expectancies. The low-risk group scored significantly lower on positive as well as negative expectancies (19.1 compared with 23.2, t = -11.8; p < 0.001, and 11.4 compared with 17.5, t = -18.3; p < 0.001). The results were equally significant for males and females. The mean score for positive expectancies was much higher compared with the mean score on negative expectancies, indicating alcohol consumption is associated with positive expectancies much more than with negative. According to these results, high-risk drinkers exceed low-risk drinkers in both negative and positive expectancies, suggesting that cognitive representation of alcohol is different in high-risk consumers. When providing brief advice to high-risk drinkers, these differences in alcohol-related expectancies should be addressed.

